# Comparison of Bracket Adhesion Failure Rates with Resin-Modified Glass Ionomer Cement Versus Conventional Resin Adhesives: A Systematic Review and Meta-Analysis of Randomized Controlled Trials

**DOI:** 10.3390/dj14060384

**Published:** 2026-06-22

**Authors:** Celalettin Noyan Sevindik, Abdul Basir Barmak, Paul Emile Rossouw, Fawad Javed

**Affiliations:** 1 Department of Orthodontics and Dentofacial Orthopedics, Eastman Institute for Oral Health, University of Rochester, Rochester, NY 14620, USA; 2 Division of Clinical Research and Biostatistics, Eastman Institute of Oral Health, University of Rochester Medical Center, Rochester, NY 14620, USA

**Keywords:** bracket failure, glass ionomer cements, resin cements, resin-modified glass ionomer, bioactive adhesive, conventional adhesive

## Abstract

**Objectives**: The aim of the present systematic review and meta-analysis is to compare bracket adhesion failure rates between resin-modified glass ionomer cement (RMGIC) and conventional resin adhesives (CRA) during fixed orthodontic treatment (OT), based on evidence from randomized controlled trials (RCTs). **Methods**: The research question is “Is there a difference in bracket adhesion failure rates between RMGIC and CRA?” The study was performed in accordance with the PRISMA guidelines. A comprehensive literature search was performed across multiple databases without time or language restrictions through February 2026. Keywords were used in different combinations using Boolean operators. Hand searching was performed and disagreements were resolved via discussion. The risk of bias (RoB) and certainty of evidence (CoE) were assessed using the Cochrane risk of bias tool and Grading of Recommendations Assessment, Development and Evaluation approach, respectively. Quantitative data synthesis was conducted using a random-effects model to calculate pooled odds ratios and 95% confidence intervals. **Results**: Seven RCTs met the inclusion criteria. Bracket failure rates ranged from 5.95% to 15.0% for RMGIC and 3.4% to 25.0% for CRA. The pooled meta-analysis revealed no statistically significant difference in bracket failure between the two adhesive types (OR = 1.00; 95% CI: 0.60 to 1.67), although substantial statistical heterogeneity was observed (I^2^ = 69.0%, *p* = 0.0065). One included trial demonstrated significantly improved retention for RMGIC when combined with a specific enamel deproteinization conditioning step prior to bonding. Three studies had a low RoB and the remaining were judged as having “some concerns”. The overall CoE was low. **Conclusions**: Based on the currently available randomized evidence, no statistically significant difference in bracket adhesion failure rates was observed between RMGIC and CRA during fixed OT. However, given the low CoE, substantial heterogeneity among studies, and relatively short follow-up periods, these findings should be interpreted with caution. Further well-designed randomized controlled trials with longer follow-up are needed to provide more definitive conclusions.

## 1. Introduction

The success of fixed orthodontic treatment (OT) largely depends on reliable bracket bonding to the enamel surface throughout therapy [1,2]. However, detachment of brackets is a frequently encountered complication during fixed OT [1]. This can lead to prolonged treatment time, increased chairside visits, and compromised treatment efficiency [3,4]. Therefore, the selection of an appropriate bracket adhesive is a critical factor in influencing the clinical success of OT. Nevertheless, the potential influence of additional factors, such as excessive shear and tensile forces generated during mastication or occlusal interferences on bracket debonding during fixed OT cannot be overlooked [5,6].

Polymer composites are broadly utilized in biomedical and dental applications, typically consisting of a resin matrix combined with filler particles. Furthermore, incorporating micro-nano fibers and ceramic particulate reinforcements has been shown to enhance the physical, mechanical, and tribological properties of these polymer matrix materials [7]. Conventional resin-based adhesives (CRA) have long been regarded as the standard material for bracket bonding due to their high bond strength and predictable clinical performance [8]. Studies [9,10] have demonstrated that resin-based adhesive systems can achieve bond strengths within the clinically acceptable range required to maintain bracket retention throughout OT. However, bracket failure rates ranging from 0.6% to over 28% have been reported when using CRA [1,5,11]. Moreover, CRA are technique sensitive, particularly due to their dependence on strict moisture control and meticulous enamel conditioning via the acid-etch technique [5]. In other words, inadequate isolation or contamination of the etched enamel surface can potentially compromise micromechanical retention, thereby increasing the risk of bracket bond failure [5]. It is also worth mentioning that CRA are non-cariostatic and non-fluoride-releasing adhesives, which limit their ability to minimize the risk of (if not prevent) enamel demineralization around orthodontic brackets (OB) [12]. This limitation has stimulated sustained interest in the development and evaluation of bioactive orthodontic bonding materials, particularly resin-modified glass ionomer cement (RMGIC), which releases fluoride over time and may help prevent enamel demineralization and the formation of white spot lesions adjacent to OB [13,14].

It has been reported that RMGIC exhibit a greater tolerance to moisture during the bonding procedure, rendering them advantageous in clinical situations where achieving optimal isolation and a completely dry operative field is challenging [15,16]. In a randomized controlled trial (RCT), Hegarty and Macfarlane [17] assessed the efficacy of bracket adhesives using RMGIC and a CRA over a one-year period. The results showed that bonding failures occurred more often with the RMGIC product than with the CRA [17]. The study [17] concluded that the CRA demonstrates superior clinical performance in bracket adhesion compared with RMGIC. However, conflicting results have also been documented. In a multicenter single-blinded RCT, Benson et al. [18] reported that although RMGIC offers advantages such as fluoride release and moisture tolerance compared with CRA, there is no statistically significant difference in bracket failure between the two adhesives [18].

Given the variability in reported outcomes and the ongoing debate regarding the comparative clinical performance of CRA and RMGIC, a systematic synthesis of the available randomized evidence is warranted. Therefore, the aim of the present systematic review and meta-analysis is to compare bracket adhesion failure rates (BAFR) between RMGIC and CRA during fixed OT based on evidence derived from randomized controlled trials (RCTs).

## 2. Materials and Methods

### 2.1. PROSPERO Registration

Prior to study initiation, the review protocol was prospectively registered with the International Prospective Register of Systematic Reviews (PROSPERO) to ensure methodological transparency, reduce duplication, and minimize the risk of reporting bias (PROSPERO; registration number: CRD420261279416).

### 2.2. Research Question

The primary research question addressed in this systematic review and meta-analysis is: “Is there a difference in bracket adhesion failure rates between RMGIC and CRA?”

### 2.3. Inclusion and Exclusion Criteria

The inclusion criteria were as follows: (a) clinical studies on patients undergoing comprehensive fixed OT; (b) parallel arm or split-mouth RCTs that compared the BAFR between RMGIC and CRA; and (c) studies evaluating patients with a fully erupted permanent dentition and a follow-up period, with no upper age restriction. Non-RCTs, in vitro studies, case reports or series, letters to the editor, commentaries or perspectives, review articles, and studies on animal models were excluded. Furthermore, studies involving ceramic brackets, restorative-grade bioactive materials, and fluoride-releasing prosthetic luting agents were also excluded.

### 2.4. Population, Intervention, Comparison, and Outcome

The present systematic review and meta-analysis followed the Patient, Intervention, Control/comparison, Outcome framework. The population (P) consisted of patients undergoing fixed OT with bonded OB. The intervention (I) included OB bonded using RMGIC. The control/comparison (C) involved brackets bonded with conventional resin adhesives (CRA). The outcome (O) of interest was the BAFR.

### 2.5. Literature Search Protocol

The literature search for the present systematic review and meta-analysis was conducted in accordance with the Preferred Reporting Items for Systematic Reviews and Meta-Analyses (PRISMA) guidelines [18]. A comprehensive literature search was performed across multiple databases without time or language restrictions through February 2026. The following electronic databases were systematically searched: PubMed/MEDLINE, Scopus, Web of Science, Cochrane Library, Embase and Google Scholar. The search strategy combined controlled vocabulary terms and free-text keywords related to orthodontic bracket bonding, bracket failure, resin-modified glass ionomer cement, and conventional resin adhesives. The search terms included combinations of the following keywords: orthodontic brackets, bracket failure, bond failure, bond survival, adhesion failure, resin-modified glass ionomer cement, RMGIC, glass ionomer, conventional resin adhesive, composite resin, and randomized controlled trial. Boolean operators were used to refine the search strategy appropriately (Supplementary Table S1). The reference lists of relevant original studies and review articles were manually screened to identify any additional eligible studies that may have been missed during the primary search. Two independent reviewers (CNS and FJ) performed the literature search and screened the titles and abstracts of all retrieved records. Full texts of potentially eligible studies were independently assessed against the inclusion and exclusion criteria. Disagreements among the investigators (CNS and FJ) were resolved through discussion, and where necessary, consultation with a third reviewer (PER) was undertaken to reach consensus.

### 2.6. Risk of Bias

Two independent investigators (CNS and FJ) assessed the risk of bias (RoB) using the revised Cochrane risk-of-bias tool for randomized trials (RoB 2) [19]. The evaluation focused on the following domains: bias arising from the randomization process, bias due to deviations from intended interventions, bias due to missing outcome data, bias in measurement of the outcome, and bias in selection of the reported result. Each domain was judged as low risk of bias, some concerns, or high risk of bias [19]. Following domain-specific evaluations, an overall RoB judgment was assigned to each study based on the highest level of bias observed across the domains. Any discrepancies between the two reviewers during the RoB assessment process were resolved through discussion and consensus. When disagreements persisted, consultation with a third reviewer (PER) was undertaken.

### 2.7. Certainty of Evidence

The certainty of evidence (CoE) for the outcomes included in the present systematic review and meta-analysis was evaluated using the Grading of Recommendations Assessment, Development and Evaluation (GRADE) approach [20]. The primary outcome was BAFR, assessed using five domains: RoB, inconsistency of results, indirectness of evidence, imprecision of effect estimates, and potential publication bias (PB). The CoE was categorized into four levels: high, moderate, low, or very low. Two reviewers (CNS and FJ) independently performed the GRADE assessment for each outcome. Disagreements were resolved through discussion and consensus. When disagreements persisted, consultation with a third reviewer (PER) was undertaken.

### 2.8. Meta-Analysis

The primary effect measure was the Odds Ratio (OR) with its corresponding 95% Confidence Interval (CI) calculated based on the raw event counts (number of bracket failures relative to the total number of brackets bonded) for both the RMGIC and conventional resin adhesive groups. This represented the overall first-time bracket failure rate (the number of first-time bracket failures relative to the initial total number of brackets bonded), rather than an overall failure rate per patient or a count of repeated failures on the same tooth. A random-effects model was used to pool data. Statistical heterogeneity among the studies was assessed and quantified using the I^2^ statistic and its corresponding *p*-value, with I^2^ values greater than 50% considered to indicate substantial heterogeneity. Additionally, visual inspection of a funnel plot was conducted to evaluate potential PB and small-study effects. The threshold for statistical significance was set at *p* < 0.05. All statistical analyses were performed using [SPSS Version 22, Chicago, IL, USA].

## 3. Results

### 3.1. Study Selection and Characteristics

**Figure 1:**
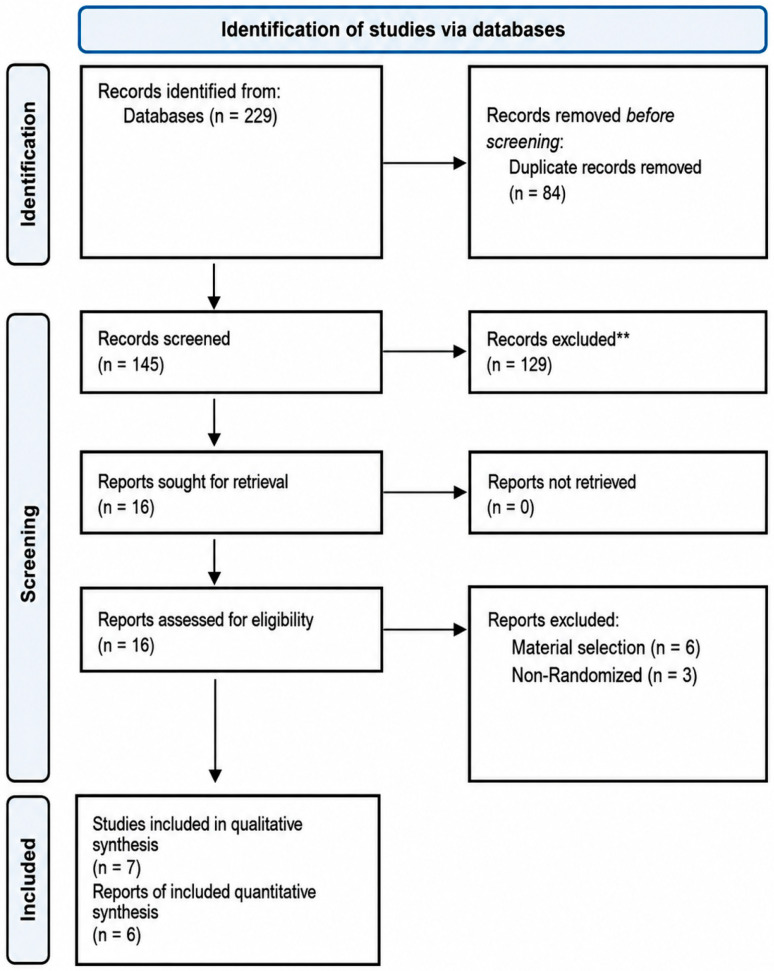
PRISMA flow diagram. ** Studies not meeting the eligibility criteria.

Two hundred and twenty-nine potentially relevant records were identified through initial database searching, with an additional 12 records found via manual backward citation screening. After removing 84 duplicates, 145 unique titles and abstracts were screened for eligibility. Of these, 129 records were excluded as they did not meet the inclusion criteria, and 16 full-texts were assessed. Following full-text review, nine non-randomized clinical investigations were excluded. In total, seven RCTs [18,19,20,21,22,23,24] were included with six [18,19,20,22,23,24] providing sufficient binary data for the quantitative meta-analysis (Figure 1). Five RCTs [19,20,21,23,24] were split-mouth randomized controlled trials and two [18,22] utilized a parallel-group design. In the included studies [18,19,20,21,22,23,24], the number of participants ranged from 20 to 197. Six studies [18,19,21,22,23,24] reported the mean ages of participants, ranging from 11 to 37 years. The clinical observation periods spanned from 6 to 18 months. The teeth evaluated generally included incisors, canines, and premolars, with most studies explicitly excluding molars from their bracket survival analyses [18,19,20,21,22,23,24] (Table 1). Prior sample size estimation (SSE) was performed in four studies [18,20,21,22,24].

### 3.2. Bracket Failures

Overall, bracket adhesion failure rates for the RMGIC and CRA groups ranged from 5.95% to 15.0% and 3.4% to 25.0%, respectively. In six RCTs [18,19,21,22,23,24], there was no statistically significant difference in the BAFR for brackets bonded with RMGIC or CRA. In the study by Ghoubril et al. [20], the BAFR was significantly higher in the CRA than the RMGIC group (Table 2).

### 3.3. Meta-Analysis

**Figure 2:**
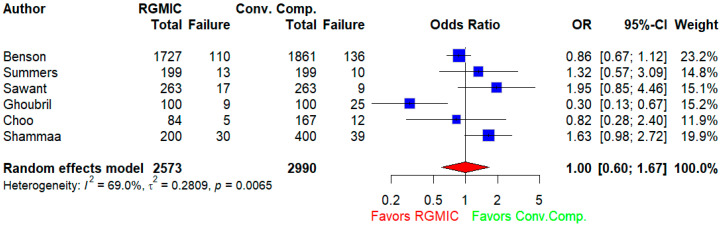
Forest plot comparing bracket adhesion failure rates between resin-modified glass ionomer cements (experimental) and conventional resin adhesives (control). The blue squares represent the effect estimate (odds ratio) from each individual study. The red diamond at the bottom represents the overall pooled effect from the random-effects meta-analysis. OR: Odds Ratio, CI: Confidence Interval, I^2^: Heterogeneity. Benson et al. [18], Summers et al. [24], Sawant et al. [22], Ghoubril et al. [20], Choo et al. [19], Shammaa et al. [23].

Six trials provided sufficient binary data and were pooled for the quantitative meta-analysis of bracket adhesion failure [18,19,20,22,23,24]. The trial conducted by Qabool et al. [21] was included in the qualitative synthesis but excluded from the quantitative meta-analysis, as the authors reported outcomes utilizing continuous survival time (mean survival days) rather than binary failure event counts. These six trials comprised a total of 5563 bonded brackets, with 2573 in the RMGIC group and 2990 in the conventional composite group. The random-effects meta-analysis revealed no statistically significant difference in bracket failure between the RMGIC adhesives and conventional composites, yielding a pooled OR of 1.00 (95% CI: 0.60 to 1.67) (Figure 2). Substantial and statistically significant heterogeneity was observed among the included trials (I^2^ = 69.0%, *p* = 0.0065) [18,19,20,22,23,24]. The weighting of the individual studies varied significantly, with the large multicenter trial by Benson et al. [18] contributing the highest weight (23.2%), while smaller trials like Choo et al. [19] contributed less (11.9%).

### 3.4. Publication Bias

**Figure 3:**
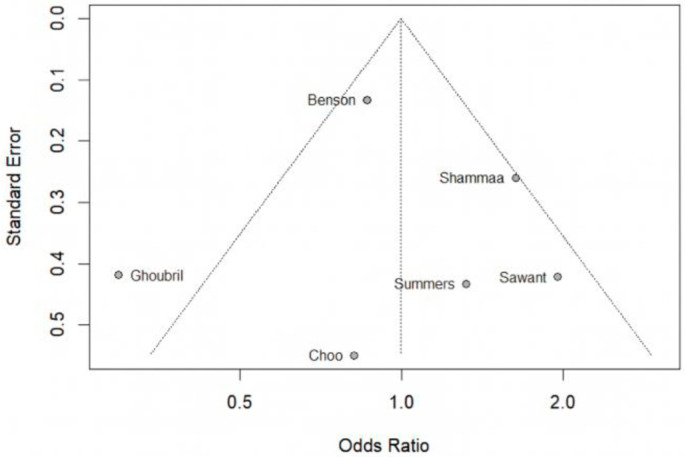
Funnel plot assessing potential publication bias and small-study effects for the included randomized controlled trials.

The funnel plot assessing the six trials [18,19,20,22,23,24] demonstrated asymmetry (Figure 3). In this plot, the standard error of the intervention effect is utilized on the y-axis as a measure of study size and precision. The distribution of the smaller trials, particularly the outlier effect of Ghoubril et al. [20] (OR = 0.30), deviates from the expected symmetrical inverted funnel shape around the pooled estimate.

### 3.5. Risk of Bias and GRADE Analyses

**Figure 4:**
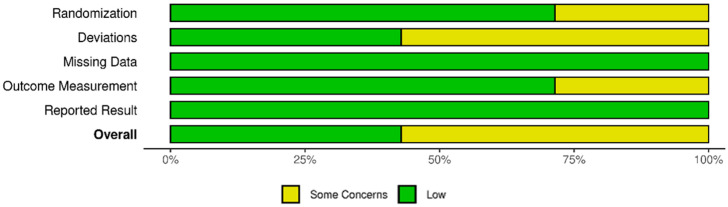
Risk of bias assessment.

Three RCTs [18,21,24] had an overall “low” RoB and the remaining [19,20,22,23] were judged to have “some concerns” (Figure 4). The CoE was rated as “low” primarily due to some concerns related to the RoB, substantial heterogeneity among the included studies (I^2^ = 69%), imprecision reflected by wide confidence intervals (OR = 1.00 [95% CI: 0.60–1.67]), and the presence of suspected PB based on funnel plot asymmetry (Table 3).

## 4. Discussion

An important methodological consideration pertains to the terminology used in the search strategy of the present systematic review and meta-analysis. The term “resin composite” is considered the scientifically preferred terminology in dental materials science [25]; however, when authors of the present study conducted supplementary searches using both terms (composite resin and resin composite) they retrieved the same body of relevant literature. Therefore, the likelihood of relevant studies being missed due to the use of the term composite resin is minimal. Nevertheless, we acknowledge that adopting the contemporary nomenclature (resin composite) in future systematic reviews may better align with current scientific standards and terminology recommendations. It is pertinent to note that in the present study, publication bias was explored through visual assessment of funnel plot symmetry. However, the interpretation of funnel plots and the application of statistical tests for funnel plot asymmetry should be undertaken with caution when a meta-analysis includes a limited number of studies [18,19,20,22,23,24]. According to the Cochrane Handbook for Systematic Reviews of Interventions, tests for funnel plot asymmetry are generally not recommended when fewer than 10 studies are available, as the statistical power of these methods is insufficient to reliably distinguish true asymmetry from chance variation [26]. This limitation represents an important methodological constraint of the current review and should be considered when interpreting the findings.

In summary, the results showed no statistically significant difference in BAFR between RMGIC and CRA [18,19,20,21,22,23,24]. This observation suggests that contemporary RMGIC formulations are capable of achieving clinically acceptable bond strengths that are comparable to those of established resin-based adhesive systems. These results are consistent with high-quality evidence, including the multicenter randomized controlled trial by Benson et al. [18], which reported comparable BAFR between the two materials. However, quantitative evaluation of the data demonstrated the presence of substantial heterogeneity (I^2^ = 69%) across the included studies [18,19,20,21,22,23,24]. This heterogeneity reflects genuine variability across studies [18,19,20,21,22,23,24] rather than random error and likely arising from differences in enamel conditioning strategies, adhesive formulations, and patient-related biomechanical factors. For instance, in studies by Choo et al. [19] and Summers et al. [24], 10% polyacrylic acid etching of enamel surface was performed to enhance chemical adhesion of RMGIC; whereas Benson et al. [18], Qabool et al. [21] and Sawant et al. [22] used 37% phosphoric acid to promote micromechanical bracket adhesion to enamel. From a mechanistic standpoint, these divergent conditioning strategies may fundamentally alter the adhesive interface, shifting the dominant bonding mechanism from primarily chemical adhesion (in the case of polyacrylic acid conditioning) to micromechanical interlocking (with phosphoric acid etching). Consequently, such procedural variations may have influenced bond strength outcomes and contributed to the observed inter-study heterogeneity. This variability limits the reliability of a universally applied pooled estimate and necessitates a cautious interpretation of the findings. Furthermore, although the seven included studies represent the entirety of the currently available randomized evidence on this topic, this small sample size restricts the power of the meta-analysis and affects the robustness of the conclusions. Collectively, these findings underscore that while RMGIC and CRA exhibit comparable overall clinical performance in bracket survival, the effectiveness of each adhesive system is highly context-dependent, influenced by operative protocols, substrate preparation, and biomechanical conditions within the oral environment.

A critical yet underreported determinant of orthodontic bracket adhesion failure is the biomechanical influence of occlusion [27]. From a biomechanical standpoint, patients presenting with bruxism and unfavorable occlusal relationships, such as deep overbite, anterior traumatic occlusion, or posterior crossbite, are exposed to increased shear and tensile forces during mastication and parafunctional activity [11,28]. It is noteworthy that approximately 57% of the RCTs [18,19,22,24] included in the present systematic review and meta-analysis did not specify the type of malocclusion being treated during orthodontic therapy. Furthermore, none of the studies included reported the presence of parafunctional habits, such as bruxism, within the study populations. In the absence of such critical clinical information, it is challenging to ascertain the true biomechanical environment to which the bonded brackets were exposed. Consequently, variations in unreported occlusal characteristics or parafunctional loading may have influenced BAFR independently of the adhesive system used, thereby confounding the interpretation of comparative outcomes between RMGIC and CRA. The authors applaud the results reported in the study by Millett et al. [27], which demonstrated that while malocclusions (such as Angle Class I vs. Class II) may not independently predict bond failure, localized occlusal interferences and functional loading patterns play a more decisive role in bracket debonding. Accordingly, it is reasonable to infer that vertical overlap, occlusal trauma, and the magnitude of masticatory forces may serve as more robust predictors of bracket adhesion failure rates than the choice of bonding adhesive alone.

In addition to occlusion, the impact of patient-specific demographics must be considered. The populations in the included studies predominantly consisted of adolescents with average ages ranging from 14 to 16 years. Within these matched cohorts, neither gender nor age variations demonstrated a statistically significant correlation with increased debonding rates. Bracket survival remains primarily a function of the biomechanical environment and operator technique rather than inherent demographic characteristics [11]. Another important observation is that molars were included only one [22] of the seven RCTs [18,19,20,21,22,23,24]. It is well established that posterior teeth, especially molars, are particularly susceptible to occlusal loading, especially in patients with deep bite or increased bite force [6,29,30]. Studies [6,31] have shown that bracket failures are disproportionately higher in regions subjected to direct occlusal interference, supporting the hypothesis that mechanical overload is a primary etiological factor. In this context, the exclusion of molars in several trials included in the present review further limits the ability to fully capture the impact of occlusal forces, as these teeth experience the highest maximum bite forces during function, averaging approximately 620 Newtons in healthy adults [32]. It is suggested that future investigations should incorporate standardized and comprehensive assessments of occlusal characteristics, including precise documentation of malocclusion type, vertical overlap, occlusal interferences, and bite force distribution. In addition, stratified or subgroup analyses based on these biomechanical parameters are warranted to delineate their independent effects on BAFR.

Over 50% of the studies [19,20,22,23] were judged as having “some concerns” related to the RoB. The principal methodological limitation was related to deviations from intended interventions, largely because complete operator blinding is inherently difficult in orthodontic bonding trials. In contrast, missing outcome data and selective reporting were generally judged to be low risk across the included studies, suggesting that attrition and outcome-reporting bias were unlikely to be major contributors to the observed findings. The GRADE analysis further contextualized these methodological findings by rating the overall CoE for BAFR as “low” [18,19,20,21,22,23,24]. This judgment was primarily driven by concerns related to RoB, substantial inconsistency, imprecision, and suspected PB. Although the pooled estimate showed no statistically significant difference between resin-modified glass ionomer cement and conventional resin adhesives, the confidence interval was wide and compatible with both potential benefit and potential harm. Moreover, the substantial heterogeneity observed across the included studies indicates that the pooled result should not be interpreted as a universal equivalence between the two adhesive systems under all clinical conditions.

The SSE is a fundamental component of robust clinical research as it determines whether a study has sufficient statistical power to detect a clinically meaningful difference if such a difference truly exists [33,34]. In the present systematic review and meta-analysis, nearly 43% of the included studies [19,23,24] were not power-adjusted, indicating that a substantial proportion of the evidence base may be vulnerable to type II error. Underpowered studies may incorrectly conclude that there is no significant difference between RMGIC and CRA when the study lacked an adequate sample size to detect a meaningful difference in BAFR. This limitation is especially important in orthodontic bracket survival research, since bracket failure is a clustered, multifactorial outcome rather than merely adhesive-dependent. The authors suggest that future RCTs should incorporate rigorous a priori sample-size calculations based on clinically meaningful differences in the BAFR. Such calculations could account for clustering at the patient level, split-mouth or parallel-arm design, anticipated attrition, expected baseline failure rates, and the intra-class correlation coefficient. Moreover, future studies could also standardize enamel-conditioning protocols, isolation methods, curing-light specifications, bracket systems, arch location, tooth type, and operator experience. It is proposed that failure outcomes should be reported using consistent definitions, preferably including both bracket-level and patient-level failure data, time-to-failure analysis, and adhesive remnant index scores. Stratified analyses according to tooth type, anterior versus posterior location, malocclusion severity, deep bite, and moisture-control difficulty may improve clinical interpretability. Finally, future trials may include longer follow-up periods and could evaluate not only bracket survival but also secondary outcomes, including enamel demineralization, white spot lesion development, chairside time, patient-centered outcomes, and cost-effectiveness.

## 5. Conclusions

Based on the currently available randomized evidence, no statistically significant difference in bracket adhesion failure rates was observed between RMGIC and CRA during fixed OT. However, given the low CoE, substantial heterogeneity among studies, and relatively short follow-up periods, these findings should be interpreted with caution. Further well-designed randomized controlled trials with longer follow-up are needed to provide more definitive conclusions.

## Figures and Tables

**Table 1:** Characteristics of included studies.

Author (Year)	RCT Design	Participants	Mean Age	Gender	Groups	Dental Malocclusion	Duration	Teeth Evaluated
Benson et al. [18]	Parallel	197	15.5 ± 3.3 years	80 M, 117 F	RMGIC vs. CRA	NR	12 months	Incisors, canines, and premolars in both arches (Molars excluded)
Choo et al. [19]	Split-Mouth	20	14.8 ± NR years	NA	RMGIC vs. CRA	NR	12 months	Incisors, canines, and premolars in both arches (Molars were banded)
Ghoubril et al. [20]	Split-Mouth	25	NR	13 M, 12 F	RMGIC vs. CRA	Class I and mild Class II	18 months	Only 1st and 2nd premolars in both arches
Qabool et al. [21]	Split-Mouth	33	16.8 ± 9.7 years	16 M, 17 F	RMGIC vs. CRA	Class I, Class II Div 1, Class II Div 2, and Class III	6 months	Incisors, canines, and premolars in both arches (Molars excluded)
Sawant et al. [22]	Split-Mouth	30	19.9 ± 4.1 years	12 M, 18 F	RMGIC vs. CRA	NR	6 months	Incisors, canines, premolars, and 1st molars
Shammaa et al. [23]	Parallel	30	16.3 ± NR years	13 M, 17 F	RMGIC vs. CRA	Class I and Class II Div 1	14 months	Incisors, canines, and premolars in both arches (Molars excluded)
Summers et al. [24]	Split-Mouth	22	14.0 ± NR years	9 M, 13 F	RMGIC vs. CRA	NR	15 months	Incisors, canines, and premolars in both arches (Molars excluded)

CRA: Conventional Resin Adhesive; NR: Not reported; F: Female; M: Male; RCT: Randomized controlled trial; RMGIC: Resin modified glass ionomer cement; NA: Not available.

**Table 2:** Bracket adhesion failure rates.

Author (Year)	RMGIC Failures/Total (n/N)	RMGIC Failure Rate (%)	CRA Failures/Total (n/N)	CRA Failure Rate (%)	*p*-Value	Key Findings
Benson et al. [18]	110/1727	6.4%	136/1861	7.3%	*p* = 0.35	No difference in failure rates.
Choo et al. [19]	5/84	5.95%	12/167	7.2%	*p* = 0.17	No difference in failure rates.
Ghoubril et al. [20]	9/100	9.0%	25/100	25.0%	*p* = 0.002	RMGIC had significantly better retention.
Qabool et al. [21]	NR	NR	NR	NR	*p* = 0.08	No difference in failure rates.
Sawant et al. [22]	17/263	6.5%	9/263	3.4%	*p* = 0.108	No difference in failure rates.
Shammaa et al. [23]	30/200	15.0%	39/400	9.8%	*p* = 0.22	No difference in failure rates.
Summers et al. [24]	13/199	6.5%	10/199	5.0%	*p* = 0.41	No difference in failure rates.

CRA: Conventional Resin Adhesive; NR: Not reported; RMGIC: Resin modified glass ionomer cement; n/N: number of failures per total number of brackets bonded.

**Table 3:** GRADE analysis.

Certainty Assessment							No. of Patients		Effect		Certainty	Importance
No. of Studies	Study Design	Risk of Bias	Inconsistency	Indirectness	Imprecision	Other Considerations	CAT	Control	Relative Effect (95% CI)	Absolute Effect (95% CI)		
6	Randomized trials	serious	serious	not serious	serious	publication bias suspected	2573	2990	OR 1.00 (0.60 to 1.67)	No clear difference	Low	CRITICAL

## Data Availability

Data is available on reasonable request.

## Supplementary Materials

The following supporting information can be downloaded at: https://www.mdpi.com/article/10.3390/dj14060384/s1, Table S1: PRISMA 2020 checklist.

## Abbreviations

The following abbreviations are used in this manuscript:

CI	Confidence Interval
CRA	Conventional resin-based adhesives
CoE	Certainty of evidence
GRADE	Grading of Recommendations Assessment, Development and Evaluation
OR	Odds ratios
OT	Orthodontic treatment
PB	Publication bias
PRISMA	Preferred Reporting Items for Systematic Reviews and Meta-Analyses
RCT	Randomized controlled trial
RCTs	Randomized controlled trials
RMGIC	Resin-modified glass ionomer cement
RoB	Risk of bias
SSE	Sample Size Estimation
